# Study on the construction of a risk assessment model for type 2 diabetes complications based on GlycA and HDL1-TC

**DOI:** 10.3389/fendo.2026.1814397

**Published:** 2026-06-08

**Authors:** Jinxin Kou, Jingjing Tie, Minjie Li, Qing Tang, Hui Sun, Zhen Wei, Yang Zhao, Ying Liu, Xianfei Zeng

**Affiliations:** 1 School of Medicine, Northwestern University, Xi’an, China; 2 Xi’an Siyuan College, Xi’an, China; 3 Laboratory Department of Xi’an Da Xing Hospital, Xi’an, China; 4 Second Affiliated Hospital of Shaanxi University of Traditional Chinese Medicine, Xi’an, China; 5 Xi’an Regional Medical Laboratory Center, Xi’an, China

**Keywords:** complications, GlycA, nuclear magnetic resonance spectroscopy, risk stratification model, type 2 diabetes

## Abstract

**Background:**

This study evaluated NMR-derived GlycA and lipoprotein subfractions for stratifying type 2 diabetes (T2DM) complication risk.

**Methods:**

A retrospective cross-sectional study included 228 adults with T2DM from two hospitals between January and December 2023. Clinical variables, the NMR-derived inflammatory markers GlycA and GlycB, and lipoprotein subfractions were analyzed. A stepwise logistic model was assessed by ROC analysis, Hosmer–Lemeshow test, bootstrap calibration, and decision-curve analysis.

**Results:**

Age, diabetes duration, fasting plasma glucose, GlycA, and HDL1-TC were retained. The model showed good fit (χ² = 7.141, P = 0.521), discrimination (AUC = 0.82, 95% CI: 0.756–0.873), calibration (MAE = 0.021), and higher net benefit than HbA1c/hs-CRP.

**Conclusions:**

A model integrating GlycA, HDL1-TC, and clinical factors showed good performance for T2DM complication risk stratification, but causal inference and temporal prediction cannot be established because of the cross-sectional design.

## 1 Introduction

The prevalence of type 2 diabetes mellitus (T2DM) is continuously increasing, and it has become a significant public health issue. The core of its long-term management lies in the prevention of complications and the control of risks (1, 2). The complications of T2DM mainly include macrovascular diseases and microvascular diseases, which are the key factors causing disability, death and increased medical burden for patients (3, 4). Due to the fact that complications often lack specific symptoms in the early stage, it is easy for clinicians to miss the optimal intervention window. Therefore, establishing a predictive tool that can be used for early risk stratification is of great significance (4).

Current research indicates that chronic low-level inflammation and lipid metabolism disorders jointly contribute to the occurrence and development of T2DM complications, and are involved in key processes such as the formation of atherosclerosis and microcirculation damage (5, 6). However, in clinical practice, the assessment of the risk of complications still mainly relies on tests such as the urine protein/creatinine ratio, imaging, and electrophysiological examinations. These tests are more used to confirm the existence and extent of complications. They still provide relatively insufficient support for comprehensive risk stratification and quantitative assessment based on test indicators (7). Glycated hemoglobin (HbA1c), although an important indicator for blood sugar control, is affected by factors such as red blood cell lifespan, pregnancy, and differences in detection methods. It mainly reflects the average blood sugar level and is unable to capture the fluctuations in blood sugar, oxidative stress, and inflammatory responses, which are closely related to the occurrence of complications. Therefore, its explanatory power for individual differences in complications is limited (8). High-sensitivity C-reactive protein (hs-CRP), as a non-specific inflammatory indicator, is greatly influenced by an individual’s physiological state, acute responses, and various exogenous factors. It is difficult to accurately and stably reflect the chronic low-level inflammation level of patients with type 2 diabetes mellitus (9, 10).

Acetylated glycoprotein (GlycA) is a composite inflammatory indicator formed by comprehensively quantifying the N-acetylglucosamine residues signals of various acute-phase glycoproteins based on nuclear magnetic resonance (NMR) spectroscopy. It has good analytical stability and repeatability (11). Compared with a single inflammatory factor, GlycA can reflect the chronic low-level inflammatory state from the perspective of the “overall protein inflammatory profile”, and has been reported to be associated with the outcomes of T2DM and atherosclerosis (12). The N-acetylated neuraminic acid glycoprotein signal B (glycoprotein acetyls B, GlycB) is another type of related glycoprotein signal indicator. Although the current research is relatively limited, it is believed that it can, to a certain extent, complement the reflection of GlycA on the systemic inflammatory state and provide additional information for the assessment of inflammatory phenotypes (13). Apart from inflammation, the heterogeneity of lipoprotein particles is closely related to residual cardiovascular risk. Traditional lipid tests are unable to reflect the differences in lipoprotein subcomponents, while NMR can conduct more precise quantification of lipoprotein subtypes, providing richer information for risk assessment (14–16). Based on the above background, this study combined the new NMR inflammatory indicators GlycA and GlycB with lipid protein subtyping indicators, and integrated conventional clinical variables to establish and internally validate a T2DM complication risk stratification model, providing a laboratory-based basis for quantitative assessment of clinical complication risks.

## 2 Methods

### 2.1 Source of the research subjects and research design

This study adopted a retrospective cross-sectional design. Patients with T2DM who visited the Second Affiliated Hospital of Shaanxi University of Chinese Medicine and Xi’an Da Xing Hospital from January 2023 to December 2023 were consecutively included as the research subjects. The outcome event of the study was “whether diabetic chronic complications occurred”, and the patients were divided into the no-complication group and the complication group; complications were further classified into macrovascular complications and microvascular complications based on clinical types. Finally, n = 228 subjects were included for analysis, and baseline information such as gender, age, and duration of diabetes were recorded (17). Inclusion criteria: Adhere to the DM diagnosis standards of the “Chinese Guidelines for the Prevention and Treatment of Type 2 Diabetes (2020 Edition)”: (1) Age ≥ 18 years; (2) Meet the T2DM diagnostic criteria; (3) Complication assessment records are completed within the study time window, and the outcome information is clear and can be determined; (4) Complete key test data: fasting blood glucose, HbA1c, hs-CRP, and NMR detection indicators; (5) Clinical data can be traced and meet the variable requirements for modeling. Exclusion criteria: (1) Type 1 diabetes, gestational diabetes, or other special types of diabetes; (2) During sampling/assessment, there are acute infections, acute inflammatory reactions, or acute cardiovascular and cerebrovascular events that may significantly interfere with inflammatory and metabolic indicators (such as acute infections, trauma, or surgery within the past 4 weeks); (3) Complicated with active malignant tumors, autoimmune disease in active stage, or long-term immunosuppressive treatment; (4) Severe liver or kidney failure or other end-stage organ failure; (5) Ineligible sample quality; (6) Missing clinical data exceeding 30%.

This retrospective study was conducted in accordance with the Declaration of Helsinki and relevant Chinese ethical requirements for biomedical research involving human participants. The study protocol was reviewed and approved by the Ethics Committee of Northwestern University (approval number: 20221216001). Because this study used previously collected clinical and laboratory data, involved no additional intervention or contact with participants, and all data were anonymized before analysis, the requirement for written informed consent was waived by the ethics committee.

### 2.2 Definition of complications and stratification criteria

The primary outcome for model construction was the presence of any chronic diabetic complication, defined as the presence of at least one macrovascular or microvascular complication. Macrovascular and microvascular complications were analyzed separately only in exploratory subgroup ROC analyses, and no separate multivariable models were constructed for these subtypes because of the limited number of events in each subgroup.

#### 2.2.1 Major vascular complications: including coronary heart disease, myocardial infarction, stroke or transient ischemic attack, and peripheral artery disease

These conditions were identified based on documented discharge diagnosis, specialist diagnosis, imaging or interventional examination records, including coronary angiography or coronary computed tomography angiography for coronary artery disease, cranial computed tomography or magnetic resonance imaging for stroke, and vascular ultrasound, computed tomography angiography, or interventional records for peripheral artery disease.

#### 2.2.2 Microvascular complications: including diabetic kidney disease, diabetic retinopathy, and diabetic peripheral neuropathy

Diabetic kidney disease was defined according to KDIGO-based criteria using albuminuria and/or reduced estimated glomerular filtration rate. Diabetic retinopathy was defined based on ophthalmologic or fundus examination records consistent with ETDRS-based grading. Diabetic peripheral neuropathy was defined according to ADA recommendations, based on documented neuropathic symptoms or signs, neurological examination, and, when available, electrophysiological findings.

#### 2.2.3 For patients with multiple complications, each complication type was recorded separately for subgroup analyses

In the overall risk stratification model, the outcome was defined as a binary variable indicating whether the patient had at least one chronic diabetic complication. To reduce potential misclassification bias, patients with incomplete, ambiguous, or unverifiable complication records were not classified as having the corresponding complication.

### 2.3 Collection of basic information and clinical data

Extract and verify the patient’s clinical baseline data and test indicators from the electronic medical record system and the laboratory information system. The collected clinical data include age, gender, duration of diabetes, family history of diabetes, history of kidney disease, history of cardiovascular and cerebrovascular diseases, whether suffering from diabetic complications, types of complications, blood pressure, fasting blood glucose. By calculating based on the patient’s height and weight: body mass index (BMI) = weight (kg)/[height (m)²].

### 2.4 Routine inspection and testing methods

#### 2.4.1 Collection and preservation of blood samples

After the subjects fasted for 10–16 hours, 3 tubes of venous blood were collected in an fasting state: 1) EDTA anticoagulation tube: used for HbA1c testing, stored at 2-8 °C and completed the test within 7 days; 2) Additive-free dry tube: after coagulation, centrifuged to separate the serum, and the serum was aliquoted into two portions: one was stored at 2-8 °C for hs-CRP testing, and the other was used for NMRS testing; 3) Sodium fluoride tube: used for fasting blood glucose testing, stored at 2-8 °C and completed the test within 8 hours.

#### 2.4.2 NMRS detection of novel inflammatory indicators and lipoprotein subcomponents

The detection was carried out using the German Bruker Avance IVDr nuclear magnetic resonance metabolic analysis system. The detection items included the novel inflammatory indicators GlycA, GlycB, and 112 lipoprotein subcomponents. After the serum samples were restored to room temperature, 400 μl of serum buffer and 400 μl of serum were added to a 2 ml centrifuge tube and manually mixed; 600 μl of the mixture was transferred to a 5 mm nuclear magnetic tube, labeled with sample information, and placed in the automatic sampler sample rack for testing. Quality control of the instrument was performed before each sample test to ensure the quality of the spectra and the comparability and accuracy of the quantitative results. After setting up the lock field and field homogenization, the automatic sampler control interface was entered. According to the experimental form, the data storage path, sample name, solvent, experiment type, sample rack number, and experiment title were set, the task was submitted, and the operation was automatically run in sequence. The data was automatically saved to the designated path. In the Bruker IVDr lipoprotein subclass analysis, HDL particles were subdivided into four HDL subclasses, namely HDL-1 to HDL-4. HDL1-TC refers to the total cholesterol content within the HDL-1 subclass, which is different from conventional total HDL-C measured in routine lipid testing.

#### 2.4.3 hs-CRP testing

The test was conducted using the immunoturbidimetric method, with the hs-CRP assay kit. The test was performed on the Beckman AU5821 automatic biochemical analyzer. The work sheet settings, sample loading, and testing were completed according to the instrument’s procedures.

#### 2.4.4 HbA1c testing

The test was conducted using high-performance liquid chromatography. The analysis was performed with the Japanese Toyoda HLC723-G11 glycosylated hemoglobin analyzer, along with the accompanying reagents and chromatographic columns.

#### 2.4.5 Fasting blood glucose test

The test was conducted using the hexokinase method on the Beckman AU5821 automatic biochemical analyzer.

### 2.5 Model construction and performance evaluation

The model performance is evaluated from two aspects: discrimination ability and calibration ability. The discrimination ability is analyzed using the ROC curve, and the AUC is calculated to assess the model’s ability to distinguish between “complications” and “no complications”. The closer the AUC value is to 1, the better the discrimination efficiency of the model is. At the same time, a ROC comparison is made with a single indicator (such as HbA1c, hs-CRP, GlycA and GlycB, etc.) to evaluate the gain of the combined model. To further demonstrate the applicability of the model in different complication spectra, stratified ROC analysis is conducted separately for major vascular complications and microvascular complications, and the AUC is calculated. Calibration was evaluated using bootstrap internal validation with 1,000 resamples. Calibration performance was assessed using the calibration curve, mean absolute error, calibration intercept, and calibration slope.

### 2.6 Statistical analysis and model construction

Statistical analysis and data processing were conducted using SPSS (version 22.0) and GraphPad Prism 10. For normally distributed measurement data, the mean ± standard deviation was used to represent them. For non-normally distributed data, the median and interquartile range [median (25th and 75th percentiles)] were used. Comparisons between the two groups were performed using t-tests or Mann-Whitney U tests. For categorical variables, chi-square tests were used for comparisons between groups. Univariate logistic regression was first performed to screen for factors associated with T2DM complications. Variables with P < 0.05 in univariate analysis were entered as candidate variables for multivariable modeling. Multivariable stepwise logistic regression was then performed using forward selection, with an entry threshold of P < 0.05 and a removal threshold of P > 0.10. No variables were forced into the model. Considering the potential correlations among GlycA, GlycB, and multiple lipoprotein subfractions, correlation patterns among candidate variables were checked before multivariable modeling. For correlated variables, the final retained predictors were determined according to their independent statistical contribution in the multivariable model and clinical interpretability. The Hosmer–Lemeshow test was used to evaluate the goodness of fit of the model. The final model was used to construct a risk stratification nomogram, which was generated using the rms package in R software. Correlation analyses between GlycA, GlycB, fasting blood glucose, glycosylated hemoglobin, and high-sensitivity C-reactive protein were performed using Spearman rank correlation.

## 3 Results

### 3.1 General characteristics of the research subjects

A total of 228 patients with T2DM were included in this study, and 6 clinical characteristics were compared (**Table 1**). The patients were divided into the complication group (161 cases) and the non-complication group (67 cases) based on whether they had diabetes complications. Compared with the non-complication group, patients in the complication group were older, had a longer duration of diabetes, and had higher fasting blood glucose and blood pressure levels (all *P* < 0.05), while there were no statistically significant differences in gender composition, body mass index (BMI), and family history of diabetes between the two groups (*P* > 0.05).

**Table 1:** Comparison of clinical data between the T2DM complication group and the non-complication group.

Characteristics	Complications	No complications	t/z/χ^2^	*p*
	(n=161)	(n=67)		
Gender [n (%)]			1.760	0.185
Male	98 (60.9)	47 (70.1)		
Female	63 (39.1)	20 (29.8)		
Family history [n (%)]			2.400	0.121
Yes	78 (48.4)	40 (61.5)		
No	83 (51.6)	27 (38.5)		
Blood pressure (mmHg)			6.313	0.012*
Normal	89 (55.3)	49 (73.1)		
Hypertension	72 (44.7)	18 (26.9)		
Course of the disease (years)	6 (3,11)	3 (1,5)	-4.925	<0.001*
Age (years)	61 (52,67)	50 (43,58)	-5.154	<0.001*
BMI (kg/m^2^)	24.96 ± 3.09	24.86 ± 2.54	0.229	0.819

P-values are unadjusted and should be interpreted as exploratory.

* indicates a statistically significant difference between the complication group and the non-complication group, with P < 0.05.

### 3.2 Comparison of laboratory indicators between the complication group and the group without complications

The fasting blood glucose level in the complication group was higher than that in the group without complications (*P* = 0.010), while there was no statistically significant difference in HbA1c and hs-CRP between the two groups (both P > 0.05). The new inflammatory indicators detected by nuclear magnetic resonance spectroscopy showed that the levels of GlycA and GlycB in the complication group were significantly increased (*P* = 0.001 and *P* = 0.026, respectively). In terms of lipoprotein subtypes, among the lipoprotein subtypes, multiple VLDL-related components were elevated and some HDL cholesterol subtypes were decreased (some indicators *P* < 0.05), suggesting that in addition to abnormal glucose metabolism, the structural redistribution of the lipoprotein particle spectrum may be related to the occurrence of complications (**Table 2**).

**Table 2:** Comparison of laboratory indicators between the T2DM complication group and the non-complication group.

Indicators	Complications	No complications	t/z	*P*
	(n=161)	(n=67)		
FPG (mmol/L)	8.70 (7.78,11.20)	7.96 (6.80,9.87)	-2.598	0.010*
HbA_1c_ (%)	8.90 (7.50,10.45)	8.93 (7.40,9.0)	-0.952	0.341
hsCRP (mg/L)	2.1 (1.10,4.20)	2.70 (1.30,4.90)	-1.583	0.113
GlycA (p.d.u)	0.87 (0.81,0.92)	0.84 (0.81,0.88)	-3.709	0.001*
GlycB (p.d.u)	0.33 (0.31,0.36)	0.33 (0.30,0.35)	-2.277	0.026*
TG (mg/dL)	128.88 (95.42,211.24)	124.39 (93.01,198.57)	-1.887	0.059
TC (mg/dL)	197.84 (160.78,223.07)	203.24 (184.37,221.50)	0.428	0.669
ApoA1 (mg/dL)	126.99 (116.23,142.32)	130.38 (111.48,149.69)	-0.755	0.450
ApoA2 (mg/dL)	29.04 (25.29,31.24)	28.78 (24.94,32.90)	-0.769	0.442
ApoB100 (mg/dL)	85.89 (69.94,106.51)	95.33 (73.25,120.54)	-0.539	0.059
LDL-C/HDL-C	2.15 (1.62,2.76)	2.46 (1.78,3.36)	-0.095	0.924
ABA1	0.69 (0.55,0.81)	0.69 (0.53,0.84)	-1.279	0.201
ApoB-P (nmol/L)	1561.68 (1277.03,1936.56)	1699.16 (1337.79,2191.77)	-0.622	0.534
VLDL-P (nmol/L)	205.67 (147.77,278.37)	181.26 (123.30,236.11)	-2.009	0.045*
IDL-P (nmol/L)	95.32 (71.46,125.42)	98.47 (80.41,115.26)	-0.979	0.328
LDL-P (nmol/L)	1208.70 (932.04,1558.08)	1449.82 (1112.06,1825.51)	-0.145	0.884
LDL1-P (nmol/L)	138.04 (90.52,203.58)	156.60 (136.43,217.12)	-1.845	0.065
LDL2-P (nmol/L)	78.91 (47.18,137.78)	83.60 (42.14,125.08)	-0.327	0.743
LDL3-P (nmol/L)	164.12 (106.22,230.88)	142.03 (119.00,241.75)	-0.261	0.794
LDL4-P (nmol/L)	265.72 (173.35,363.09)	284.03 (174.77,359.28)	0.287	0.774
LDL5-P (nmol/L)	271.91 (185.75,353.84)	248.63 (192.55,389.86)	-1.026	0.305
LDL6-P (nmol/L)	345.88 (239.26,480.68)	396.88 (213.47,541.85)	-1.118	0.264
VLDL-TG (mg/dL)	96.17 (63.18,155.14)	90.52 (51.98,147.48)	-1.973	0.049*
IDL-TG (mg/dL)	9.82 (5.37,19.94)	7.06 (5.30,19.82)	-1.782	0.075
LDL-TG (mg/dL)	21.13 (15.11,25.07)	21.80 (18.60,27.94)	-0.12	0.904
HDL-TG (mg/dL)	10.03 (6.63,15.24)	10.00 (8.81,14.83)	-0.115	0.909
VLDL-TC (mg/dL)	25.31 (17.29,41.16)	22.97 (16.57,36.51)	-1.811	0.070
IDL-TC (mg/dL)	13.80 (9.69,19.92)	12.72 (10.64,17.91)	-1.864	0.062
LDL-TC (mg/dL)	90.18 (65.68,123.29)	105.86 (79.09,146.06)	-1.051	0.293
HDL-TC (mg/dL)	41.93 ± 9.84	45.15 ± 13.90	-2.352	0.049*
VLDL-FC (mg/dL)	11.42 (7.62,17.49)	9.71 (5.58,16.17)	-1.978	0.048*
IDL-FC (mg/dL)	4.10 (2.86,5.84)	3.62 (3.19,5.59)	-1.702	0.089
LDL-FC (mg/dL)	33.00 (25.31,40.98)	33.85 (25.93,46.15)	-0.576	0.564
HDL-FC (mg/dL)	12.94 (10.80,14.45)	11.73 (10.26,15.39)	-0.501	0.616
VLDL-PL (mg/dL)	30.64 ± 17.33	23.21 ± 15.90	-2.303	0.021*
IDL-PL (mg/dL)	7.94 (5.69,12.95)	8.04 (5.77,11.40)	-1.181	0.237
LDL-PL (mg/dL)	54.43 (41.89,71.12)	61.72 (49.16,79.74)	-1.009	0.313
HDL-PL (mg/dL)	60.26 (53.55,71.28)	65.37 (49.12,76.71)	-1.298	0.197
HDL-ApoA1 (mg/dL)	117.44 (103.22,130.69)	113.71 (98.61,133.45)	-0.425	0.671
HDL-ApoA2	29.28 (25.82,31.49)	29.13 (25.85,34.05)	-0.217	0.828
VLDL-ApoB100 (nmol/L)	11.31 (8.18,15.72)	9.49 (6.46,12.99)	-1.993	0.046*
IDL-ApoB100 (nmol/L)	5.24 (3.93,6.90)	5.18 (4.08,6.27)	-1.186	0.236
LDL-ApoB100 (nmol/L)	66.48 (51.26,85.69)	79.74 (61.16,100.40)	-0.145	0.884
VLDL1-TG (mg/dL)	38.21 (23.21,69.63)	39.03 (17.89,58.66)	-1.648	0.099
VLDL2-TG (mg/dL)	19.05 (11.75,29.28)	16.76 (6.64,22.96)	-2.272	0.023*
VLDL3-TG (mg/dL)	17.44 (11.89,25.89)	14.59 (11.03,18.14)	-2.892	0.004*
VLDL4-TG (mg/dL)	11.38 (7.83,15.4)	8.40 (6.7,13.66)	-2.599	0.009*
VLDL5-TG (mg/dL)	2.55 (1.7,3.24)	2.53 (2.20,3.27)	-0.331	0.756
VLDL-1TC (mg/dL)	7.16 (3.86,13.46)	5.75 (2.44,12.70)	-1.871	0.061
VLDL-2TC (mg/dL)	4.34 (2.59,7.69)	3.74 (1.72,5.47)	-2.76	0.006*
VLDL-3TC (mg/dL)	6.12 (3.80,9.65)	5.20 (2.52,6.14)	-3.337	0.001*
VLDL-4TC (mg/dL)	6.62 (4.08,8.43)	4.79 (3.47,6.78)	-3.271	0.001*
VLDL-5TC (mg/dL)	1.29 (0.75,2.13)	1.07 (0.69,1.89)	-1.685	0.092
VLDL-1FC (mg/dL)	2.47 (1.19,4.7)	1.72 (0.93,4.45)	-1.827	0.068
VLDL-2FC (mg/dL)	2.15 (1.09,3.47)	1.91 (0.70,3.12)	-1.527	0.127
VLDL-3FC (mg/dL)	2.72 (1.58,4.53)	2.22 (0.94,2.94)	-2.267	0.023*
VLDL-4FC (mg/dL)	3.15 (1.86,4.19)	2.18 (1.75,3.35)	-2.310	0.021*
VLDL-5FC (mg/dL)	0.28 (0.00,0.66)	0.07 (0.00,0.39)	-1.975	0.048*
VLDL1-PL (mg/dL)	6.78 (4.13,12.15)	7.33 (3.54,16.01)	-0.881	0.379
VLDL2-PL (mg/dL)	5.05 (2.93,7.63)	4.65 (2.77,6.75)	-1.866	0.062
VLDL3-PL (mg/dL)	5.69 (3.81,8.16)	4.74 (3.13,6.41)	-2.573	0.010*
VLDL4-PL (mg/dL)	5.62 (4.10,7.51)	4.62 (3.97,5.64)	-2.518	0.012*
VLDL5-PL (mg/dL)	1.72 (1.13,2.38)	1.56 (1.22,2.28)	-1.297	0.195
LDL1-TG (mg/dL)	4.54 (2.42,6.98)	4.92 (3.84,6.98)	-0.369	0.712
LDL2-TG (mg/dL)	1.77 (1.22,2.47)	1.88 (1.25,2.37)	-0.301	0.772
LDL3-TG (mg/dL)	1.80 (1.28,2.38)	1.86 (1.52,2.59)	0.28	0.709
LDL4-TG (mg/dL)	3.54 ± 1.43	3.29 ± 1.39	1.214	0.227
LDL5-TG (mg/dL)	3.60 (2.72,5.22)	3.23 (2.43,4.94)	-1.634	0.102
LDL6-TG (mg/dL)	4.94 (4.06,6.22)	4.75 (3.31,6.36)	-1.796	0.072
LDL1-TC (mg/dL)	14.23 (9.18,21.72)	14.03 (11.68,21.82)	-0.603	0.547
LDL2-TC (mg/dL)	3.01 (0.00,8.09)	4.99 (0.01,14.42)	-1.321	0.186
LDL3-TC (mg/dL)	14.12 (8.27,19.86)	13.70 (10.03,22.71)	-0.596	0.551
LDL4-TC (mg/dL)	21.37 (12.76,30.87)	22.71 (12.99,28.88)	0.015	0.988
LDL5-TC (mg/dL)	20.16 (13.96,27.19)	20.98 (14.94,29.95)	-0.658	0.511
LDL6-TC (mg/dL)	23.07 (16.01,30.60)	26.25 (13.70,35.37)	-0.927	0.354
LDL1-FC (mg/dL)	4.30 (3.13,6.97)	5.01 (3.69,6.87)	-1.003	0.316
LDL2-FC (mg/dL)	0.54 (0.00,2.79)	0.94 (0.00,7.00)	-1.351	0.177
LDL3-FC (mg/dL)	4.91 ± 2.40	5.26 ± 2.24	-0.946	0.344
LDL4-FC (mg/dL)	5.99 ± 2.67	6.37 ± 2.61	0.017	0.986
LDL5-FC (mg/dL)	6.01 (4.24,7.40)	6.23 (4.69,8.40)	-0.998	0.319
LDL6-FC (mg/dL)	5.91 (4.36,7.77)	6.75 (3.93,9.37)	-0.173	0.863
LDL1-PL (mg/dL)	8.06 (5.17,12.42)	8.65 (7.72,12.37)	-1.251	0.211
LDL2-PL (mg/dL)	1.24 (0.00,4.75)	2.17 (0.00,7.32)	-1.415	0.157
LDL3-PL (mg/dL)	8.20 (5.22,12.10)	7.93 (5.99,11.91)	-0.878	0.380
LDL4-PL (mg/dL)	12.45 ± 5.48	11.91 ± 5.83	0.665	0.507
LDL5-PL (mg/dL)	11.06 (7.98,14.72)	11.00 (8.49,16.51)	-0.726	0.468
LDL6-PL (mg/dL)	13.13 (9.48,17.04)	15.26 (8.77,19.57)	-0.774	0.439
LDL1-ApoB (mg/dL)	7.65 (5.00,12.18)	8.61 (7.50,11.93)	-1.234	0.217
LDL2-ApoB (mg/dL)	0.33 (0.00,3.19)	2.88 (0.00,10.44)	-1.402	0.161
LDL3-ApoB (mg/dL)	9.32 (5.84,13.18)	8.97 (6.80,14.18)	-0.713	0.476
LDL4-ApoB (mg/dL)	14.42 (9.43,19.60)	15.67 (9.68,19.76)	-0.469	0.639
LDL5-ApoB (mg/dL)	14.95 (10.23,19.46)	13.67 (10.59,21.44)	-1.028	0.304
LDL6-ApoB (mg/dL)	19.02 (13.16,26.44)	21.83 (11.74,29.8)	-1.118	0.264
HDL1-TG (mg/dL)	2.84 (1.05,4.73)	3.26 (2.10,4.78)	-0.654	0.513
HDL2-TG (mg/dL)	1.52 (0.68,2.38)	1.65 (1.12,2.53)	-0.389	0.697
HDL3-TG (mg/dL)	1.48 (0.66,2.32)	1.52 (0.87,2.39)	-0.536	0.592
HDL4-TG (mg/dL)	3.65 (3.04,4.98)	3.46 (2.77,4.52)	-1.836	0.066
HDL1-TC (mg/dL)	9.56 (6.72,14.83)	14.8 (9.01,20.7)	-2.776	0.010*
HDL2-TC (mg/dL)	5.48 (4.03,7.39)	6.62 (4.70,8.81)	-2.332	0.020*
HDL3-TC (mg/dL)	7.68 (6.42,9.34)	7.84 (7.09,9.96)	-2.397	0.017*
HDL4-TC (mg/dL)	18.69 ± 5.53	18.33 ± 6.28	0.425	0.671
HDL1-FC (mg/dL)	3.83 (3.33,4.46)	4.46 (3.37,5.91)	-2.852	0.013*
HDL2-FC (mg/dL)	1.71 (1.50,2.22)	1.84 (1.45,2.53)	-2.263	0.024*
HDL3-FC (mg/dL)	1.90 (1.50,2.33)	1.79 (1.37,2.29)	-0.36	0.719
HDL4-FC (mg/dL)	4.46 ± 1.46	4.12 ± 1.54	-1.909	0.056
HDL1-PL (mg/dL)	11.92 (8.35,18.10)	16.95 (9.87,22.42)	-1.831	0.067
HDL2-PL (mg/dL)	8.32 (5.97,12.15)	9.31 (8.02,13.62)	-1.989	0.047*
HDL3-PL (mg/dL)	11.86 (10.02,14.13)	12.23 (9.70,15.39)	-1.512	0.131
HDL4-PL (mg/dL)	26.77 ± 6.28	25.22 ± 7.53	1.594	0.112
HDL1-ApoA1 (mg/dL)	10.53 (5.90,18.22)	14.91 (6.68,22.80)	-0.86	0.390
HDL2-ApoA1 (mg/dL)	12.39 (10.03,14.83)	12.83 (9.74,17.12)	-1.737	0.082
HDL3-ApoA1 (mg/dL)	20.67 (17.38,23.86)	19.32 (15.99,25.94)	-1.311	0.190
HDL1-ApoA2 (mg/dL)	1.41 (0.73,2.34)	1.64 (1.22,2.51)	-0.865	0.387
HDL2-ApoA2 (mg/dL)	1.91 (1.24,2.61)	2.19 (1.73,2.70)	-2.073	0.038*
HDL3-ApoA2 (mg/dL)	5.08 (4.42,5.84)	5.29 (4.61,7.14)	-1.177	0.239
HDL4-ApoA2 (mg/dL)	19.67 ± 4.73	19.16 ± 4.72	0.728	0.467

* indicates a statistically significant difference between the complication group and the non-complication group, with P < 0.05.

### 3.3 Univariate Logistic Regression Analysis of Complications of Type 2 Diabetes Mellitus

The indicators with statistically significant differences in the single-factor comparison were included in the single-factor Logistic regression analysis (**Table 3**). The results showed that age, duration of diabetes, fasting blood glucose, blood pressure, new inflammatory indicators GlycA and GlycB, as well as multiple subcomponents of VLDL-related lipoproteins (including VLDL3-TG, VLDL4-TG, VLDL2-TC, VLDL3-TC, VLDL4-TC, etc.) were all risk factors for T2DM complications (OR > 1, P < 0.05). On the contrary, multiple HDL-related subcomponents (HDL1-TC, HDL2-TC, HDL3-TC, HDL1-FC, HDL2-FC, and HDL2-ApoA2) were protective factors (OR < 1, P < 0.05), suggesting that the changes in the lipoprotein particle profile may have supplementary value in the risk stratification of complications.

**Table 3:** Univariate logistic regression between the T2DM complication group and the non-complication group.

Indicators	Coef	OR	95% confidence interval	*P*
Age (years)	0.075	1.078	1.047-1.110	<0.001***
Course of disease (years)	0.147	1.158	1.079-1.242	<0.001***
FPG (mmol/L)	0.147	1.159	1.029-1.305	0.015*
Blood pressure (mmHg)	0.789	2.202	1.181-4.107	0.013*
GlycA (p.d.u)	0.647	1.910	1.290-2.828	0.001**
GlycB (p.d.u)	1.085	2.958	1.249-7.008	0.014*
VLDL3-TG (mg/dL)	0.033	1.034	1.006-1.063	0.017*
VLDL4-TG (mg/dL)	0.068	1.071	1.015-1.130	0.013*
VLDL2-TC (mg/dL)	0.103	1.108	1.022-1.202	0.013*
VLDL3-TC (mg/dL)	0.119	1.126	1.044-1.241	0.002**
VLDL4-TC (mg/dL)	0.126	1.135	1.039-1.239	0.005*
VLDL3-PL (mg/dL)	0.094	1.099	1.007-1.199	0.034*
VLDL4-PL (mg/dL)	0.127	1.135	1.008-1.278	0.036*
HDL1-TC (mg/dL)	-0.068	0.934	0.898-0.972	0.001*
HDL2-TC (mg/dL)	-0.117	0.889	0.808-0.979	0.017*
HDL3-TC (mg/dL)	-0.202	0.817	0.729-0.916	0.001*
HDL1-FC (mg/dL)	-0.336	0.714	0.588-0.868	0.011*
HDL2-FC (mg/dL)	-0.599	0.542	0.362-0.833	0.005*
HDL2-ApoA2 (mg/dL)	-0.339	0.712	0.558-0.909	0.006*

“*” indicates P < 0.05, “**” indicates P < 0.01, and “***” indicates P < 0.001.

### 3.4 Establishment of a risk stratification model for complications of type 2 diabetes mellitus

The variables that were significantly correlated in the univariate Logistic regression were included in the stepwise Logistic regression model, with whether to combine T2DM complications as the dependent variable (**Table 4**). The results of the multivariate analysis showed that the duration of diabetes, age, fasting blood glucose, and the new inflammatory indicator GlycA were independent risk factors for T2DM complications (all *P* < 0.05), while HDL1-TC was an independent protective factor (*P* < 0.05). Based on these variables, a T2DM complication risk stratification model was constructed, and its regression equation was: Logit(P) = −8.786 + 0.107 × duration of diabetes + 0.064 × age + 0.166 × fasting blood glucose + 0.595 × GlycA − 0.082 × HDL1-TC. The Hosmer–Lemeshow test results showed that the model fit well (χ² = 7.141, P = 0.521). The Nagelkerke R² of the final model was 0.355, indicating that the model had a certain explanatory ability for T2DM complications. Although GlycB was significantly associated with T2DM complications in univariate logistic regression analysis (OR = 2.958, 95% CI: 1.249–7.008, P = 0.014), it was not retained in the final multivariable stepwise model. After accounting for the retained predictors, GlycB did not meet the predefined entry criterion, suggesting that its additional independent contribution was limited in the present model.

**Table 4:** Establishment of Risk Stratification Model for T2DM Complications.

Indicators	Coef	SD	Waldχ^2^	*P*	OR	95% confidence interval
Course of disease (years)	0.107	0.041	6.979	0.008	1.113	1.028-1.206
Age (years)	0.064	0.018	12.089	0.001	1.066	1.028-1.105
FPG (mmol/L)	0.166	0.074	5.069	0.024	1.180	1.022-1.363
GlycA (p.d.u)	0.595	0.235	6.422	0.011	1.813	1.144-2.873
HDL1-TC (mg/dL)	-0.082	0.024	11.352	0.001	0.921	0.878-0.966
Constant	-8.786	2.305	14.522	0.000	0.000	

Hosmer–Lemeshow χ² = 7.141, P = 0.521; Nagelkerke R² = 0.355. HDL1-TC indicates the total cholesterol content in the HDL-1 subclass measured by NMR-based lipoprotein subclass analysis.

### 3.5 Evaluation of independent factors and discriminative performance of the risk stratification model

**Figure 1:**
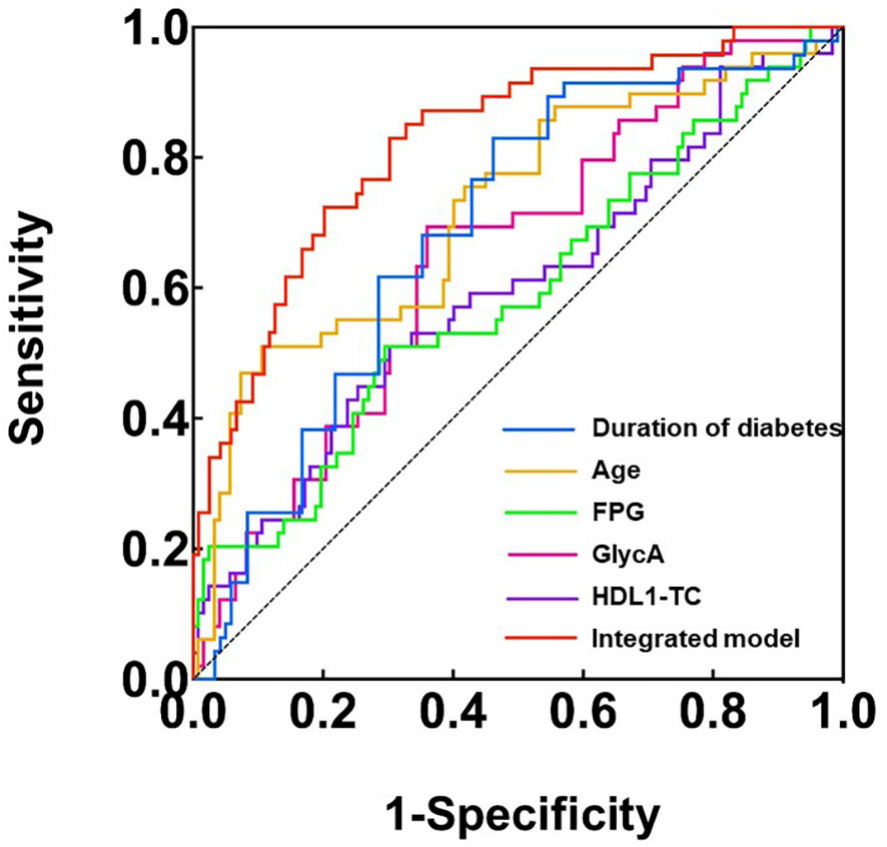
Compares the discriminatory ability of diabetes duration, age, FPG, GlycA, HDL1-TC and the integrated risk stratification model for T2DM complications. The x-axis represents 1-specificity; the y-axis represents sensitivity.

The ROC curve analysis showed that each individual independent risk factor had discriminatory ability for identifying T2DM complications: the AUCs of diabetes duration, age, new inflammatory indicators GlycA and HDL1-TC were 0.71, 0.72, 0.65 and 0.61 respectively (all *P* < 0.05) (**Table 5**). The area under the ROC curve of the risk stratification model constructed based on multiple factors was 0.82, the optimal cut-off point was 0.79, corresponding to a sensitivity of 60.0% and a specificity of 91.0%, suggesting that this model has good discriminatory ability (**Figure 1**).

**Table 5:** Evaluation of predictive indicators and models for T2DM complications and their diagnostic efficacy.

Indicators	AUC	Sensitivity (%)	Specificity (%)	Optimal cutoff point	95% confidence interval	*p*
Course of disease (years)	0.71	45.1	85.1	6.50	0.637-0.777	<0.001
Age (years)	0.72	88.8	44.8	46.50	0.642-0.791	<0.001
FPG (mmol/L)	0.62	70.8	56.7	8.13	0.531-0.689	0.006
GlycA(p.d.u)	0.65	60.1	73.1	0.86	0.573-0.725	<0.001
HDL1-TC (mg/dL)	0.61	32.3	46.3	12.45	0.526-0.692	0.010
Integrated model	0.82	60.0	91.0	0.79	0.756-0.873	<0.001

### 3.6 Evaluation of the diagnostic efficacy of HbA1c and hs-CRP in complications of type 2 diabetes mellitus

**Figure 2:**
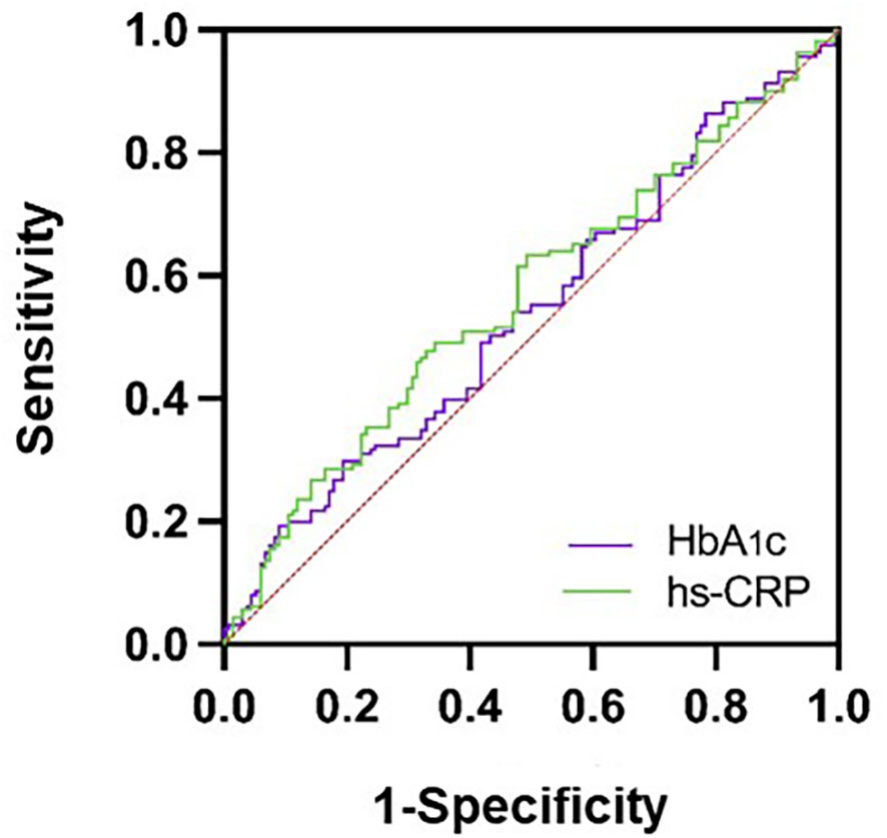
ROC curves of HbA1c and hs-CRP.

The ROC curve was used to evaluate the diagnostic efficacy of HbA1c and hs-CRP in T2DM complications. The results showed that the AUC of HbA1c was 0.54, with the optimal cut-off point being 11.15%; the AUC of hs-CRP was 0.57, with the optimal cut-off point being 2.85 mg/dL. The results indicated that there was no statistically significant difference between the AUC of HbA1c and hs-CRP and 0.5 (*P*> 0.05) (**Table 6**; **Figure 2**).

**Table 6:** Evaluation of diagnostic efficacy of HbA1c and hs-CRP in complications of type 2 diabetes mellitus.

Indicators	AUC	Sensitivity (%)	Specificity (%)	Optimal cutoff point	95% confidence interval	*p*
HbA_1c_ (%)	0.54	19.3	91.0	11.15	0.459-0.621	0.354
hs-CRP (mg/L)	0.57	47.8	67.2	2.85	0.487-0.646	0.114

### 3.7 Construction of the nomogram and model calibration

**Figure 3:**
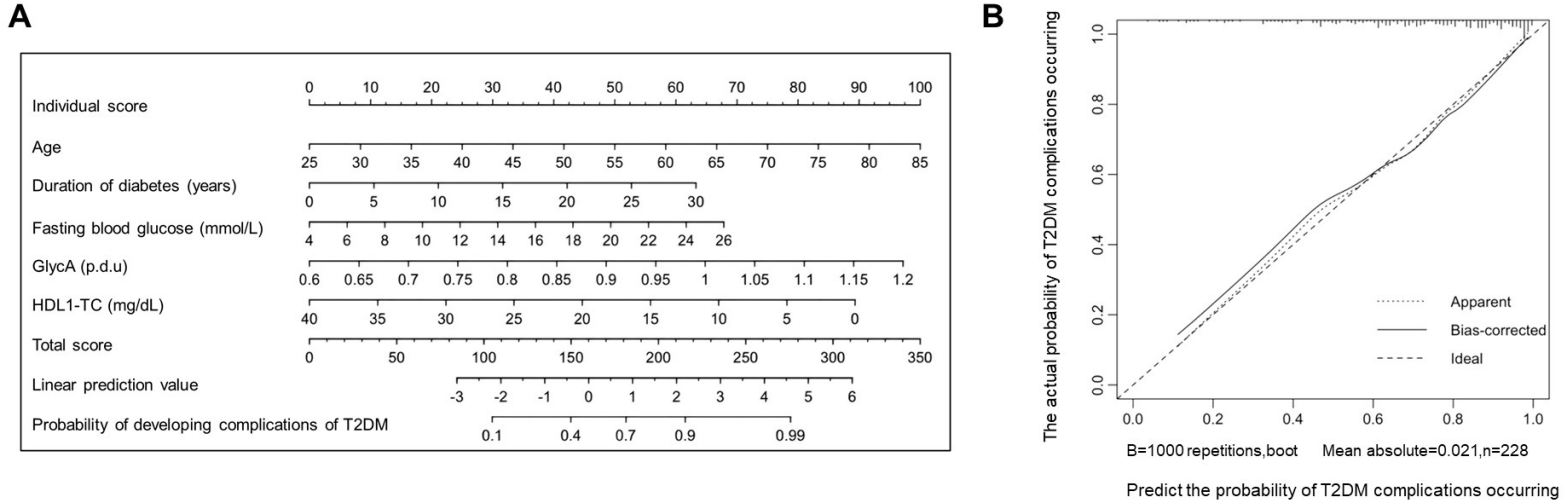
Risk prediction nomogram and model calibration for T2DM complications. **(A)** The nomogram includes point scales for age, diabetes duration, FPG, GlycA, and HDL1-TC, as well as total points and the corresponding predicted probability of T2DM complications. **(B)** The calibration curve obtained through 1,000 bootstrap resamples showed agreement between predicted and observed probabilities, with a mean absolute error of 0.021, a calibration intercept of 0.059, and a calibration slope of 0.901.

Based on the final multivariate Logistic regression model, a risk stratification nomogram for T2DM complications was further constructed. Five variables, including duration of diabetes, age, fasting blood glucose, GlycA, and HDL1-TC, were incorporated into the model. Each variable in the nomogram corresponds to a certain score, and the higher the cumulative total score, the greater the risk of complications (**Figure 3A**). To verify the robustness of the model, bootstrap internal validation with 1,000 resamples was performed. The mean absolute error (MAE) of the calibration curve was 0.021. The bootstrap-corrected calibration intercept was 0.059, and the calibration slope was 0.901. These results indicated that the predicted probabilities were generally consistent with the observed probabilities, with no obvious systematic overestimation or underestimation. The calibration curve also showed that the bias-corrected curve was close to the ideal curve, suggesting good calibration performance of the nomogram (**Figure 3B**).

### 3.8 Diagnostic efficacy of novel inflammatory indicators in different types of complications

**Figure 4:**
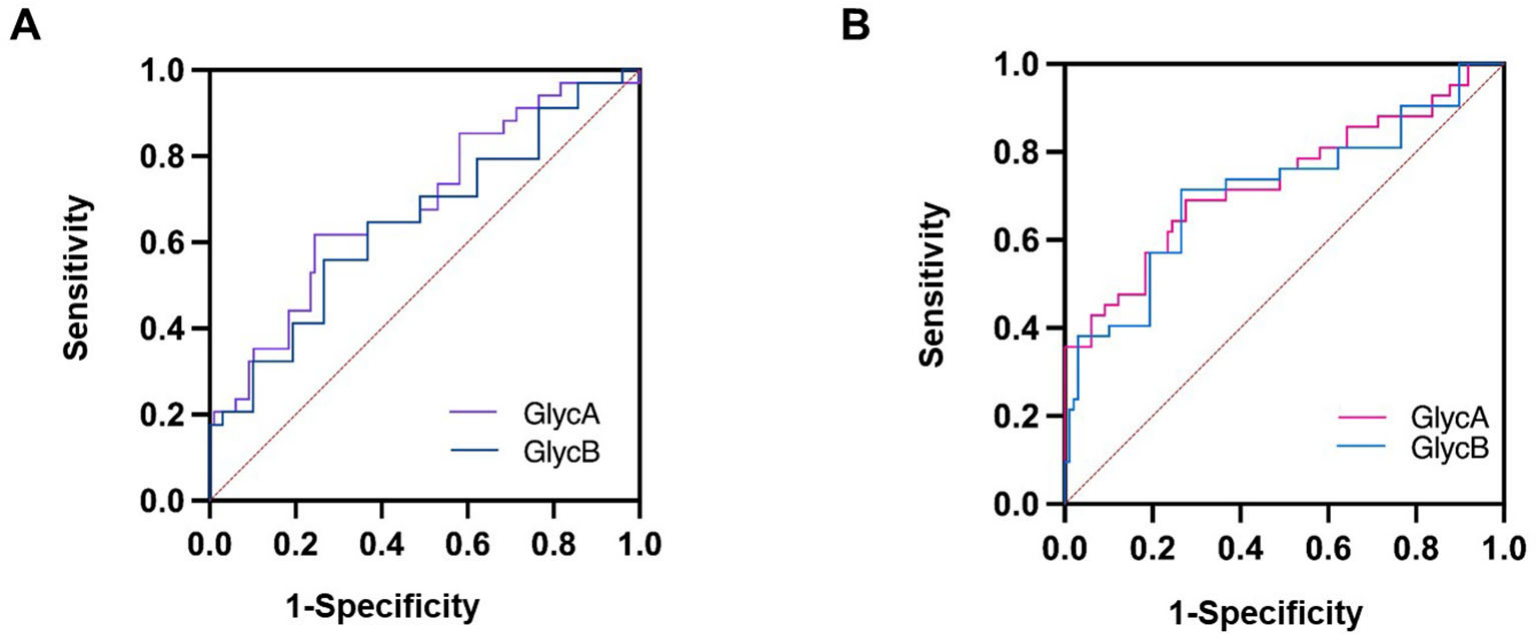
ROC curves of GlycA and GlycB for different types of complications in T2DM. **(A)** Predicting macrovascular complications; **(B)** Predicting microvascular complications. The ROC curve is used to evaluate the discriminatory ability of GlycA and GlycB. The larger the AUC, the better the discrimination.

Exploratory subgroup ROC analyses were performed to evaluate the discriminatory ability of GlycA and GlycB for different complication types. This study included 64 patients with macrovascular complications and 82 patients with microvascular complications. For macrovascular complications, the AUCs of GlycA and GlycB were 0.69 and 0.65, respectively (both P < 0.05) (**Table 7**). For microvascular complications, the AUCs of GlycA and GlycB were 0.73 and 0.71, respectively (both P < 0.001) (**Table 8**). These findings suggest that GlycA and GlycB showed slightly better discriminatory performance for microvascular complications than for macrovascular complications. However, these subgroup results should be interpreted as exploratory because separate multivariable models were not constructed for each complication subtype due to the limited number of events in each subgroup. The ROC curves showed that GlycA had slightly higher discriminatory ability than GlycB for both macrovascular and microvascular complications (**Figures 4A, B**).

**Table 7:** ROC curve analysis of the value of GlycA and GlycB in predicting macrovascular complications of type 2 Diabetes Mellitus.

Indicators	AUC	*P*	Sensitivity (%)	Specificity (%)	Optimal cutoff point	95% confidence interval
GlycA (p.d.u)	0.69	0.004	0.62	0.76	0.86	0.57-0.80
GlycB (p.d.u)	0.65	0.024	0.56	0.69	0.33	0.52-0.77

**Table 8:** ROC curve analysis of the value of GlycA and GlycB in predicting microvascular complications of type 2 diabetes mellitus.

Indicators	AUC	*P*	Sensitivity (%)	Specificity (%)	Optimal cutoff point	95% confidence interval
GlycA (p.d.u)	0.73	<0.001	0.64	0.76	0.86	0.63-0.84
GlycB (p.d.u)	0.71	<0.001	0.71	0.69	0.33	0.60-0.82

### 3.9 Decision-curve analysis of the integrated model, HbA1c, and hs-CRP for stratifying T2DM complication risk

**Figure 5:**
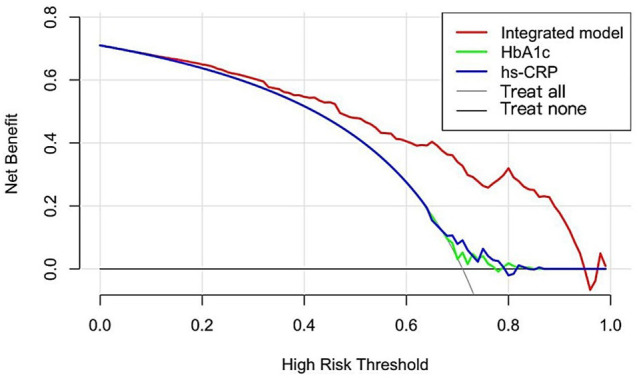
Decision-curve analysis of the integrated model, HbA1c, and hs-CRP for predicting T2DM complications. Decision-curve analysis showed the net benefit of the integrated model, HbA1c, and hs-CRP across different threshold probabilities. “Treat all” assumes that all patients are classified as high risk, whereas “treat none” assumes that no patients are classified as high risk.

Decision-curve analysis was performed to further evaluate the potential clinical usefulness of the integrated model. The results showed that the integrated model had a higher net benefit than HbA1c, hs-CRP, treat-all, and treat-none strategies across a broad range of threshold probabilities. This finding suggests that the integrated model may have potential clinical value for risk stratification of T2DM complications (**Figure 5**).

## 4 Discussion

Based on the novel inflammatory indicators GlycA and GlycB obtained through NMR and the information on lipoprotein subtypes, this study constructed a risk stratification model for T2DM complications. The main findings include: The levels of GlycA and GlycB in the complication group were significantly higher, while the traditional inflammatory indicators hs-CRP and glycosylated hemoglobin HbA1c showed no significant differences between the two groups; the lipoprotein particle profile presented the characteristic of “increased VLDL-related lipid components and decreased HDL-related cholesterol subtypes”; multivariate Logistic regression indicated that the duration of diabetes, age, and fasting blood glucose were independent risk factors for T2DM complications, and HDL1-TC was an independent protective factor. The integrated model showed good discrimination and calibration performance, suggesting its potential application value in the early clinical risk stratification.

Patients with T2DM remain in a state of high blood sugar for a long time. Long-term high blood sugar can trigger low-level inflammation and oxidative stress, which in turn promotes the release of inflammatory-related factors by immune cells and vascular endothelial cells. These signals act through the circulation on the liver, promoting an increase in the synthesis and secretion of acute-phase proteins (18, 19). GlycA, as a composite inflammatory indicator, can simultaneously reflect the changes in the N-glycan branches of various acute-phase proteins, thereby more stably characterizing systemic low-grade inflammation and disease progression (20). Previous studies have indicated that higher plasma GlycA levels are associated with an increased risk of microvascular complications in type 2 diabetes mellitus (21), and is closely related to the atherosclerotic-related phenotypes (22). This study further confirmed that GlycA is an independent risk factor for T2DM complications, suggesting that it may have significant biological significance in the development of complications and is consistent with the previous results that GlycA can be used for risk stratification and can improve diagnostic efficacy when incorporated into multi-index models (12).

In contrast, hs-CRP is considered to potentially be associated with microvascular complications of type 2 diabetes mellitus (23), however, this study did not observe a significant association between it and the risk of complications. Previous evidence regarding the relationship between hs-CRP and diabetic retinopathy has also been inconsistent, possibly due to its inability to reflect specific stages or being influenced by confounding factors such as BMI and insulin sensitivity (24, 25). HbA1c is also affected by factors such as red blood cell lifespan, pregnancy, race, age, and differences in detection methodologies. In some populations, its interpretation of the risk of complications is limited (26, 27). Previous studies have shown that patients with the same HbA1c level do not have consistent risk of complications, and their predictive efficacy for complications is limited. Even in some studies, the discriminative power of the models constructed based on HbA1c is relatively weak (8, 28). The association of HbA1c in predicting the risk of T2DM complications is not significant, which is similar to previous studies. This suggests that it is more suitable as a monitoring indicator for basal metabolism or inflammatory status rather than serving as the core discriminant variable for risk stratification of complications alone. Therefore, composite inflammatory indicators and detailed lipoprotein subclass information may provide complementary information for risk stratification of T2DM complications. It should be noted that the poor performance of HbA1c and hs-CRP in this cohort may have somewhat exaggerated the significant advantages of GlycA and HDL1-TC. Therefore, the result that the comprehensive model performed better should be understood as evidence of the potential complementary value in this dataset, rather than a clear demonstration of significant clinical value beyond traditional indicators.

In terms of model performance, this study combined GlycA with traditional risk factors and lipoprotein subcomponent indicators and showed higher observed discriminative performance than single conventional markers in this dataset that only included conventional inflammation and traditional lipid items. This suggests that lipoprotein subtyping may provide supplementary information beyond traditional lipids (29, 30). Currently, GlycB has received relatively little research in the field of T2DM complications. However, it is related to sialic acid signaling, and the association between sialic acid levels and cardiovascular diseases as well as mortality outcomes has been reported (31). This study suggests that GlycB has a certain ability to distinguish between major vascular complications and may serve as a supplementary marker.

The value of lipidomics particle profiles in cardiovascular risk assessment has been recognized. Particle indicators such as LDL-P are superior to traditional LDL-C in some situations, and lipidomics reveals new disease-related lipid characteristics (32, 33). In this study, the complication group showed a spectrum change characterized by elevated VLDL-related lipid components, suggesting a more severe triglyceride-enriched lipoprotein load. This change is consistent with the previously described mechanism of diabetic dyslipidemia: insulin resistance can promote the increase in VLDL production and secretion in the liver, thereby driving the increase in the load of atherosclerosis-related lipoprotein remnants (34). At the same time, we observed that HDL1-TC decreased and was an independent protective factor in the multivariate model; previous evidence suggests that the anti-atherosclerotic effect of HDL is mainly related to its functions of promoting cholesterol excretion or reverse transport, indicating that HDL subtype information may provide supplementary information beyond traditional lipid levels for risk stratification of complications (35). Overall, the systematic analysis of lipoprotein subcomponents is helpful in identifying potential markers for T2DM complications and improving risk stratification. However, in the present model, HDL1-TC was retained as an independent protective factor. HDL1-TC represents the total cholesterol content in the HDL-1 subclass rather than conventional total HDL-C. Therefore, this finding suggests that specific HDL subclass information may provide additional risk stratification value beyond routinely measured HDL-C. Because the biological functions of different NMR-defined HDL subclasses may vary and are not completely interchangeable with conventional HDL subfractions, the clinical interpretation of HDL1-TC should remain cautious and requires further validation.

From the perspective of clinical implementation, the cost and accessibility of NMR-based testing should be considered. Compared with routine laboratory markers such as HbA1c, fasting plasma glucose, and hs-CRP, NMR testing requires specialized instrumentation and may have higher testing costs, which may limit its use as a universal screening tool for all patients with T2DM. However, a single NMR-based assay can simultaneously provide GlycA, GlycB, and detailed lipoprotein subclass information, thereby offering a broader inflammatory and lipid-metabolic profile. Therefore, the potential clinical value of this approach may lie in its use as an adjunctive risk stratification tool for selected patients, particularly those with intermediate risk, discordant conventional markers, or a need for more detailed metabolic assessment. Further health-economic evaluation is needed to determine whether the additional information provided by NMR testing justifies its cost in routine clinical practice.

Although this study constructed a T2DM complication risk stratification model with certain discriminatory ability based on NMR-derived inflammatory indicators and lipoprotein subtypes, and showed good calibration performance in internal validation, several issues should be considered when interpreting these findings. In the present study, the integrated model showed good discrimination and calibration, and decision-curve analysis further indicated that the model had a higher net benefit than HbA1c and hs-CRP across a broad range of threshold probabilities. These findings suggest that the combination of GlycA, HDL1-TC, and clinical variables may have potential value for risk stratification of T2DM complications. However, the ROC-derived cutoff of the integrated model had high specificity (91.0%) but moderate sensitivity (60.0%). This suggests that the model may be more suitable for identifying patients with a high probability of complications rather than serving as a stand-alone screening tool. In screening settings, a lower threshold may be considered to improve sensitivity, although this would reduce specificity and increase the number of patients requiring further evaluation. Therefore, a more appropriate clinical threshold should be determined in future studies by combining decision-curve analysis, external validation, and the clinical consequences of missed diagnoses and false-positive classifications.

Several limitations should also be acknowledged. First, this was a retrospective study with a limited sample size, which may have introduced selection bias. Because of the cross-sectional design, causal relationships between GlycA, HDL1-TC, and T2DM complications cannot be inferred, and the model should be interpreted as a risk stratification model rather than a tool for predicting future incident complications. Second, although patients were recruited from two hospitals, the study population was limited to a Chinese regional medical setting, which may restrict the generalizability of the findings to other ethnic groups, regions, and healthcare systems. In addition, external validation was not performed, and the clinical utility and generalizability of the model need to be further confirmed in larger multicenter cohorts. Third, although standardized diagnostic criteria were used to define macrovascular and microvascular complications, the classification of complications depended on available medical records and examination reports. Therefore, potential misclassification bias cannot be completely excluded. To minimize this risk, patients with incomplete or ambiguous complication records were excluded from the corresponding outcome classification. Future prospective studies with predefined complication assessment protocols are needed to further validate the model. Fourth, the evidence related to GlycB remains relatively insufficient, and its stability and biological significance in stratifying T2DM complication risk still need to be verified by prospective studies. In the future, longitudinal follow-up data should be used to evaluate the relationship between dynamic changes in GlycA and GlycB and the occurrence of complications, as well as their potential application in clinical risk assessment. It should also be noted that the primary model in this study was developed for the presence of any chronic diabetic complication, rather than for a specific complication subtype. Macrovascular and microvascular complications may differ in pathophysiology, and our subgroup ROC analyses suggested that GlycA and GlycB showed slightly better discrimination for microvascular complications. However, because the number of events in the macrovascular and microvascular subgroups was limited, we did not construct separate multivariable models for each subtype to avoid unstable estimates and overfitting. Future studies with larger samples should develop and validate subtype-specific models for macrovascular and microvascular complications.

## Data Availability

The original contributions presented in the study are included in the article/supplementary material. Further inquiries can be directed to the corresponding author.
